# Properties, Structures, and Physiological Roles of Three Types of Anion Channels Molecularly Identified in the 2010’s

**DOI:** 10.3389/fphys.2021.805148

**Published:** 2021-12-23

**Authors:** Yasunobu Okada, Ravshan Z. Sabirov, Petr G. Merzlyak, Tomohiro Numata, Kaori Sato-Numata

**Affiliations:** ^1^National Institute for Physiological Sciences (NIPS), Okazaki, Japan; ^2^Department of Physiology, School of Medicine, Aichi Medical University, Nagakute, Japan; ^3^Department of Physiology, Kyoto Prefectural University of Medicine, Kyoto, Japan; ^4^Cardiovascular Research Institute, Yokohama City University, Yokohama, Japan; ^5^Laboratory of Molecular Physiology, Institute of Biophysics and Biochemistry, National University of Uzbekistan, Tashkent, Uzbekistan; ^6^Department of Integrative Physiology, Graduate School of Medicine, Akita University, Akita, Japan; ^7^Japan Society for the Promotion of Science, Tokyo, Japan

**Keywords:** volume-related anion channels, LRRC8A, SLCO2A1, TMEM206, TRPM7, cell swelling, acidosis, regulatory volume decrease

## Abstract

Molecular identification was, at last, successfully accomplished for three types of anion channels that are all implicated in cell volume regulation/dysregulation. LRRC8A *plus* LRRC8C/D/E, SLCO2A1, and TMEM206 were shown to be the core or pore-forming molecules of the volume-sensitive outwardly rectifying anion channel (VSOR) also called the volume-regulated anion channel (VRAC), the large-conductance maxi-anion channel (Maxi-Cl), and the acid-sensitive outwardly rectifying anion channel (ASOR) also called the proton-activated anion channel (PAC) in 2014, 2017, and 2019, respectively. More recently in 2020 and 2021, we have identified the S100A10-annexin A2 complex and TRPM7 as the regulatory proteins for Maxi-Cl and VSOR/VRAC, respectively. In this review article, we summarize their biophysical and structural properties as well as their physiological roles by comparing with each other on the basis of their molecular insights. We also point out unsolved important issues to be elucidated soon in the future.

## Introduction

According to the activation mechanisms, mammalian anion channels have been classified into six major groups: voltage-gated, ligand-gated receptor-coupled, Ca^2+^-activated, cAMP-activated, volume-activated, and acid-activated ones. Among them, the volume- or swelling-activated and the acid- or proton-activated anion channels, that are not directly gated by voltage, ligands, Ca^2+^, and cAMP, are known to be ubiquitously expressed and tightly involved in cell volume regulation/dysregulation (see Review: Okada et al., 2019a) and cell death induction/protection (see Reviews: Okada et al., 2021a for the former channel; Okada et al., 2021b for the latter channel). Volume-activated anion channels include two members: the volume-sensitive outwardly rectifying anion channel (VSOR), also called the volume-regulated anion channel (VRAC), and the large-conductance maxi-anion channel (Maxi-Cl). VSOR/VRAC and Maxi-Cl were functionally discovered in 1988 (Cahalan and Lewis, 1988; Hazama and Okada, 1988) and 1983 (Blatz and Magleby, 1983), respectively. The acid-sensitive outwardly rectifying anion channel (ASOR), also called the proton-activated anion channel (PAC), represents the acid-activated anion channel, and was functionally discovered in 2003 (Auzanneau et al., 2003). The core molecules for VSOR/VRAC, Maxi-Cl, and ASOR/PAC were identified at last in 2014–2019 (see below) all by unbiased genome-wide approaches, whereas molecular entities of other types of anion channels were elucidated much earlier. Here we concisely review their molecular identities, functional properties, structural features and physiological roles in comparison with each other.

## Molecular Identification for the Core and Regulatory Molecules of Volume-Sensitive Outwardly Rectifying Anion Channel/Volume-Regulated Anion Channel, Maxi-Anion Channel, and Acid-Sensitive Outwardly Rectifying/Proton-Activated Anion Channel

### The Core and Regulatory Molecules of Volume-Sensitive Outwardly Rectifying Anion Channel/Volume-Regulated Anion Channel

Leucine-rich repeat-containing eight family member A (LRRC8A) was identified as the core molecule of VSOR/VRAC in 2014 (Qiu et al., 2014; Voss et al., 2014) and was shown to form a heterohexamer with LRRC8C, 8D, and/or 8E (Voss et al., 2014; Syeda et al., 2016). Given heterogeneity of heterohexameric composition of the core component, the term VSOR/VRAC is likely to comprise a group of similar channels with a common molecular architecture and common fundamental properties of whole-cell currents (see below) but different subtype compositions in different tissues. The pore-forming role of LRRC8A was suggested by some mutation studies (see Review: König and Stauber, 2019), especially by a slight change in the P_*I*_/P_*Cl*_ ratio induced by a charge-reversing K98E mutation (Ullrich et al., 2016) and by a small increase in the Na^+^ permeability induced by a charge-neutralizing R103A mutation (Deneka et al., 2018). Also, it is noted that purified LRRC8A *plus* LRRC8C/D/E were found to be sufficient to form VSOR/VRAC channels activated by low ionic strength, though not by swelling, when reconstituted in lipid droplets by Syeda et al. (2016), the fact of which strongly suggests that the heteromer *per se* provides the pore of VSOR/VRAC channels activated by reduced ionic strength. However, whether LRRC8A *plus* 8C/D/E form the pore of swelling-activated VSOR/VRAC by themselves still awaits further study to obtain such decisive evidence as the transformation from anion selectivity to cation selectivity caused by introduction of some charge-modifying mutation at the selectivity filter site.

Most recently TRPM7 was shown to serve as an essential regulator of VSOR activity and plasmalemmal expression of LRRC8A with exhibiting the physical protein-protein interaction between LRRC8A and TRPM7 as evidenced by the effects of TRPM7 gene deletion and silencing on LRRC8A expression, stable LRRC8A expression to the plasma membrane, as well as plasmalemmal co-localization and co-immunoprecipitation between LRRC8A and TRPM7 tagged with fluorescent proteins, which were detected by antibodies specific to tagged proteins, in a manner sensitive to the deletion of the kinase domain of TRPM7 (Numata et al., 2021). To certify the general role of TRPM7 in VSOR/VRAC regulation, future studies are awaited to be performed by using different types of antibodies in a number of cell types, including those derived from TRPM7-knockout mice. Given that the LRR motif serves as the site for protein-protein interactions, it is likely that some other regulatory components for VSOR/VRAC/LRRC8 channels are still missing.

### The Core and Regulatory Molecules of Maxi-Anion Channel and Acid-Sensitive Outwardly Rectifying/Proton-Activated Anion Channel

SLCO2A1, which is known as a prostaglandin transporter (Kanai et al., 1995), was identified as the pore molecule of Maxi-Cl in 2017 (Sabirov et al., 2017). This identification extends the list of transporter-associated anion channels (see Review: Minor, 2017) such as glutamate transporter-associated anion channels, SLC1As/EAATs (see Review: Fahlke et al., 2016; Untiet et al., 2017; Engels et al., 2021) involved in chloride homeostasis. More recently in 2019, the core molecule of ASOR/PAC was identified to be TMEM206 (Ullrich et al., 2019; Yang et al., 2019a). The firmest evidence for pore-forming roles in Maxi-Cl and ASOR/PAC was provided by the observations that a charge-neutralizing K613G mutant of SLCO2A1 (Sabirov et al., 2017) and a charge-reversing K319E mutant of TMEM206 (Ruan et al., 2020) converted their activities from anion-selective to cation-selective channels, respectively.

The hetero-tetrameric S100A10-annexin A2 (ANXA2) complex formed by two S100A10 and two ANXA2 molecules was identified by Islam et al. (2020) as the regulatory component of Maxi-Cl. The physical protein-protein interaction between SLCO2A1 and ANXA2 was evidenced by co-immunoprecipitation assays. Furthermore, the ANXA2-S100A10 complex was shown to be responsible for the dependence of Maxi-Cl activity on cytosolic Ca^2+^ and tyrosine dephosphorylation (Islam et al., 2020). However, the regulatory subunit for ASOR/PAC/TMEM206 remains to be identified.

These established molecular components of VSOR/VRAC, Maxi-Cl, and ASOR/PAC are listed in Table 1A.

**TABLE 1:** Comparisons of the molecular, biophysical, and physiological properties among volume-sensitive outwardly rectifying anion channel/volume-regulated anion channel (VSOR/VRAC), maxi-anion channel (Maxi-Cl), and acid-sensitive outwardly rectifying/proton-activated anion channel (ASOR/PAC).

Anion channels	VSOR/VRAC	Maxi-Cl	ASOR/PAC
**(A) Molecular identities**			
Core	LRRC8A + 8C/D/E	SLCO2A1	TMEM206
Regulatory	TRPM7	ANXA2 + S100A10	?
**(B) Biophysical properties**			
Pore radius	∼*0*.*63* nm	0.75-1.3 nm	?
Unitary conductance	Intermediate (10-90 pS)	Large (300-500 pS)	Small (4-10 pS)
Rectification	Mild outward	Linear sharp outward	Sharp outward
Gating	Inactivation kinetics at +V*	Inactivation kinetics at +V and -V*	Activation kinetics at +V*
**(C) Physiological properties**			
Activation factors			
Strong acidity	Suppressing	?	Activating
Cell swelling	Activating	Activating	Insensitive
ROS	Activating	?	?
Regulatory factors			
Cytosolic ATP	Dependent	Inhibiting	Independent
Cytosolic Mg^2+^	Sensitive	?	Insensitive
Cytosolic Ca^2+^	Indirectly dependent	Directly dependent	Insensitive

*+V and -V represent positive and negative voltages, respectively.

## Functional Properties of Volume-Sensitive Outwardly Rectifying Anion Channel/Volume-Regulated Anion Channel, Maxi-Anion Channel, and Acid-Sensitive Outwardly Rectifying/Proton-Activated Anion Channel

Electrophysiological properties of these three types of volume-related anion channels were directly studied by observing their channel currents using patch-clamp techniques, and those were described in detail so far in the review articles for VSOR/VRAC (Strange et al., 1996; Nilius et al., 1997; Okada, 1997), Maxi-Cl (Sabirov et al., 2016, 2021), and ASOR/PAC (Okada et al., 2021b). The pharmacological properties of these three types of volume-related anion channels are different from each other, as recently summarized in our review article (Okada et al., 2019b). Here, we summarize biophysical and physiological properties of these three types of anion channels together by comparing them with each other in order to shed light on the research subjects remaining to be studied.

### Biophysical Properties of Volume-Sensitive Outwardly Rectifying Anion Channel/Volume-Regulated Anion Channel, Maxi-Anion Channel, and Acid-Sensitive Outwardly Rectifying/Proton-Activated Anion Channel

The pore radii of VSOR/VRAC and Maxi-Cl were functionally evaluated to be around 0.63 (Ternovsky et al., 2004) and 0.75–1.3 nm (Sabirov and Okada, 2004), respectively. However, the pore radius of ASOR/PAC has not been directly studied as yet. In accord with the above pore size, the unitary (single-channel) conductance of Maxi-Cl (300–500 pS) is larger than that of VSOR/VRAC (10–90 pS). Since the unitary conductance of ASOR/PAC (4–10 pS) is smaller than VSOR/VRAC, the pore radius of ASOR/PAC could be predicted to be smaller than that of VSOR/VRAC. Whole-cell currents (*I*) of these three types of anion channels are also distinct from each other in their rectification and voltage (*V*)-dependent gating time courses. The *I–V* relationship of Maxi-Cl is linear (ohmic) and distinct from those of VSOR/VRAC and ASOR/PAC exhibiting weakly and sharply outward rectifying voltage dependence, respectively. The voltage- and time-dependent activation kinetics of ASOR/PAC currents showing in response to application of positive voltages is distinct from inactivation kinetics of VSOR/VRAC currents upon positive voltages and of Maxi-Cl currents upon positive and negative voltages. These biophysical properties are listed in comparison to each other in Table 1B.

### Physiological Properties of Volume-Sensitive Outwardly Rectifying Anion Channel/Volume-Regulated Anion Channel, Maxi-Anion Channel, and Acid-Sensitive Outwardly Rectifying/Proton-Activated Anion Channel

Extracellular acidification activates ASOR/PAC but rather suppresses VSOR/VRAC (Sabirov et al., 2000). Osmotic cell swelling is a well-known stimulus not only for VSOR/VRAC but also for Maxi-Cl, whereas ASOR/PAC was found to be unaffected by osmotic cell swelling (Kittl et al., 2019). VSOR/VRAC activity is activated also by reactive oxygen species (ROS) even in the absence of cell swelling (Browe and Baumgarten, 2004; Shimizu et al., 2004; Varela et al., 2004), presumably via co-localization and physical interaction between LRRC8A and NOX1 (Choi et al., 2016) as well as co-localization and not only physical but also functional interactions between LRRC8C and NOX1 (Choi et al., 2021), although these interactions must be reexamined by using antibodies more specific to LRRC8A, LRRC8C, and NOX1. There has, however, been no study on the effects of ROS on ASOR/PAC and Maxi-Cl activities. The presence of ATP in the cytosol is definitely indispensable for VSOR/VRAC activation (Jackson et al., 1994; Oiki et al., 1994) but dispensable for ASOR/PAC activation (Yamamoto and Ehara, 2006), whereas cytosolic ATP rather downregulates Maxi-Cl activity (Sabirov and Okada, 2009). Cytosolic free Mg^2+^ was shown to reduce VSOR/VRAC activity (Oiki et al., 1994) in a concentration-dependent manner (Okada et al., 2019b) but to have no effect on ASOR/PAC (Lambert and Oberwinkler, 2005), whereas the effect of cytosolic Mg^2+^ on Maxi-Cl was not studied as yet. An increased intracellular free Ca^2+^ concentration ([Ca^2+^]_*i*_) was suggested to be somehow involved in activation of Maxi-Cl currents based on the effect of a Ca^2+^ ionophore (Light et al., 1990; Kawahara and Takuwa, 1991) and was actually shown to upregulate Maxi-Cl activity in a concentration-dependent manner by binding to S100A10 (Islam et al., 2020), whereas Ca^2+^-dependent activation of VSOR/VRAC is indirectly produced in response to stimulation of G protein-coupled receptor (GPCR; Mongin and Kimelberg, 2005) through NOX-mediated ROS production (Akita et al., 2011; Akita and Okada, 2011) at very high [Ca^2+^]_*i*_ regions in the immediate vicinity of the channel, called Ca^2+^ nanodomains (Akita and Okada, 2011). In contrast, ASOR/PAC activity is totally insensitive to cytosolic Ca^2+^ (Nobles et al., 2004; Yamamoto and Ehara, 2006; Kittl et al., 2019). These physiological properties are listed with comparing them to each other in Table 1C.

## Structural Features of Volume-Sensitive Outwardly Rectifying Anion Channel/Volume-Regulated Anion Channel, Maxi-Anion Channel, and Acid-Sensitive Outwardly Rectifying/Proton-Activated Anion Channel

### Structures of the Core Molecules of Volume-Sensitive Outwardly Rectifying Anion Channel/Volume-Regulated Anion Channel

**FIGURE 1:**
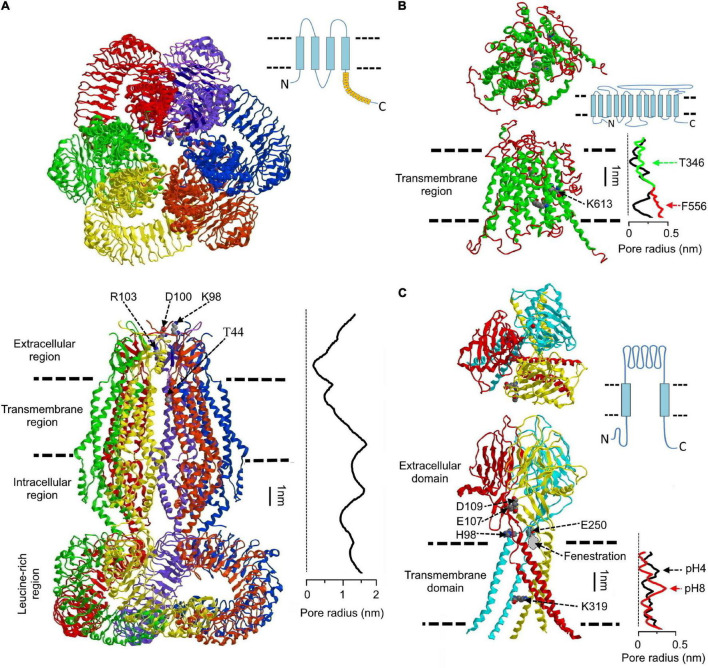
Structural features of volume-sensitive outwardly rectifying anion channel/volume-regulated anion channel (VSOR/VRAC), maxi-anion channel (Maxi-Cl), and acid-sensitive outwardly rectifying/proton-activated anion channel (ASOR/PAC). **(A)** Structure of the human homohexameric LRRC8A channel. *Upper panel*: top view; *lower panel*: side view. The pore radius along the central axis (graph at the right of the *lower panel*) is shown in scale. The structure is drawn according to Kasuya et al. (2018) using the 5zsu.pdb file downloaded from https://www.rcsb.org/structure/5zsu. On the *upper right side*, schematic membrane topology of the monomeric protein is drawn according to Voss et al. (2014). **(B)** The presumed structure of the Maxi-Cl. The homology model of the SLCO2A protein built using the glycerol-3-phosphate transporter from *Escherichia coli* as a template is shown [modified from Sabirov et al. (2017)]. On the *middle right side*, the protein membrane topology is drawn according to Nakanishi et al. (2021). The pore radius along the central pore axis (graph at the *right of the lower panel*) is calculated using HOLE program (Smart et al., 1996). Arrows indicate position of the two putative gates. Green and red lines illustrate a shift in the pore radius when indicated amino acids are replaced with Gly (see text for details). **(C)** Top (*upper panel*) and side (*lower panel*) views of the human ASOR/PAC channel. E250 of cognate subunit together with E107 and D109 of the next adjacent subunit form an “acidic pocket” for the protonated H98. The pore radius along the central axis is shown in scale. Red and black lines correspond to the deprotonated and protonated channels, respectively. The drawing is based on the 7jna.pdb file downloaded from https://www.rcsb.org/structure/7jna [Modified from Okada et al. (2021b)]. Schematic membrane topology of the monomeric protein is drawn on the *middle right side*, according to Ullrich et al. (2019).

The series of three-dimensional (3D)-structures of homohexamers of LRRC8A (Deneka et al., 2018; Kasuya et al., 2018; Kefauver et al., 2018; Kern et al., 2019) and LRRC8D (Nakamura et al., 2020) were an impressive breakthrough achieved during the past 4 years by employing cryo-EM in combination with single particle computational analysis. In the homo-hexameric assembly of human LRRC8A (Kasuya et al., 2018), single subunits have very similar topology, all of which are characterized by the following four regions: the transmembrane region (∼4 nm) is formed by transmembrane helixes (TM1–TM4); two extracellular loops between TM1 and TM2 (EL1) and TM3 and TM4 (EL2) form the extracellular region (∼3.5 nm); TM2 and TM3 are connected by an intracellular loop (IL1), and IL1 together with another intracellular loop (IL2) forms the intracellular region (∼3.5 nm); the IL2 connects TM4 with the leucine-rich repeats (LRR) region (∼6 nm) containing 15 LRRs. In the hexameric channel, the six LRRs are twisted in a clockwise manner. The overall shape and relative dimensions of the four regions of the LRRC8A homohexameric complex are illustrated in Figure 1A. Other reported structures share similar topology (Deneka et al., 2018; Kefauver et al., 2018; Kern et al., 2019). However, it must be noted that the 3D-structures of heteromeric hexamers of LRRC8A *plus* LRRC8C/D/E remain undetermined.

All four regions of the LRRC8A hexamer contribute to the channel pore, which appears to be fairly long. In the human channel (Kasuya et al., 2018), the distance from the extracellular entrance (with radius *R* ∼ 0.74 nm) to the intracellular exit vestibule (*R* ∼ 1.13 nm) is about 14 nm (Figure 1A). The pore is not uniform in size exhibiting a constriction of *R* ∼ 0.38 nm (presumably a selectivity filter) followed by a local widening with *R* ∼ 2.54 nm around the transmembrane region. Although the vestibules’ constriction and middle widening were observed in all reported 3D-structures of recombinant LRRC8 proteins, their dimensions were quite variable. Thus, the narrowest selectivity filter (*R* ∼ 0.1 nm) was observed for human LRRC8A hexamer by Kefauver et al. (2018), whereas the homohexameric channel made of human LRRC8D had the widest pore (*R* ∼ 0.57 nm) at the constriction site (Nakamura et al., 2020). The latter value is close to the size of native VSOR/VRAC: *R* ∼ 0.57–0.71 nm indirectly estimated by permeability to organic anions (Nilius et al., 1999; Nilius and Droogmans, 2003), and by blocking effects of calixarenes (Droogmans et al., 1998, 1999) and *R* ∼ 0.63 evaluated by non-electrolyte partitioning (Ternovsky et al., 2004). Obviously, experimental conditions during sample preparation, such as lipidic environment and ionic strength, significantly affect the tightness of the resulting protein assembly [see Figure 1 in Okada et al. (2021a) for summary of the pore radii at the constriction site for the published 3D-structures].

In the LRRC8A hexameric channel, the narrowest constriction site corresponds to the ring of positively charged R103 located at the *N*-terminal part of the EL1 (Deneka et al., 2018; Kasuya et al., 2018; Kefauver et al., 2018; Kern et al., 2019). This residue is a putative determinant of anion selectivity, extracellular ATP blockage and binding to a VSOR/VRAC selective blocker, 4-(2-butyl-6,7-dichloro-2-cyclopentyl-indan-1-on-5-yl) oxobutyric acid (DCPIB), that binds to the pore in a “cork-in-bottle” manner (Kern et al., 2019). T44 at the extracellular end of TM1 is also known to induce small changes in the channel selectivity to I^–^ and Cl^–^ (Qiu et al., 2014). Residues K98 and D100 located at the *C*-terminus of the EL1 are also conceived to be involved in selectivity as well as in voltage-dependent gating (Ullrich et al., 2016; Deneka et al., 2018; Kasuya et al., 2018; Kefauver et al., 2018; Strange et al., 2019). Locations of the mentioned amino acids are depicted in Figure 1A.

The uniquely arranged LRR region contains numerous charged amino acids at the contacting surfaces between adjacent subunits (Deneka et al., 2018; Kasuya et al., 2018; Kefauver et al., 2018; Kern et al., 2019; Nakamura et al., 2020). Such arrangements may explain the well-known activation by low ionic strength of the native VSOR/VRAC (Cannon et al., 1998; Voets et al., 1999; Sabirov et al., 2000; Deneka et al., 2021) and purified LRRC8 proteins in lipid bilayers (Syeda et al., 2016). This is thought to occur due to altered electrostatic interactions between subunits via a mechanism, which could also be caused by mechanical stress (Strange et al., 2019). Consistent with this paradigm, a recent study demonstrated that synthetic proteinaceous nanobodies (called sybodies) targeting different LRR epitopes profoundly modulate activation of the native channel by low ionic strength (Deneka et al., 2021).

### Structural Features of the Core Molecule of Maxi-Anion Channel

The molecular basis of the Maxi-Cl has been established only recently as the SLCO2A1 protein (Sabirov et al., 2017), and thus no high-resolution 3D-structures are available at present. Figure 1B shows the homology model of SLCO2A1 protein based on the published crystal structure of the glycerol-3-phosphate transporter (Sabirov et al., 2017). The full-length protein possesses 12 transmembrane domains, is located mostly within the membrane (Transmembrane region in Figure 1B) with relatively small extra- and intra-cellular regions. The pore radius calculated along the central axes shows two constrictions around T346 and F556 (Figure 1B: black line in the graph given at the right of the lower panel), which supposedly correspond to two gates necessary for its function as a prostaglandin transporter (Minor, 2017). Upon changing these amino acids to Gly, the modeled pore widens (Figure 1B: green line for T346G and red line for F556G). However, such widening (up to *R* ∼ 0.2–0.4 nm) in this conformation of SLCO2A1 would not allow passage of ATP, ADP, and UTP [*R* ∼ 0.53–0.61 nm; see Table 2 in Sabirov and Okada (2005)], although native Maxi-Cl channels are known to be ATP-conductive, as noted below. Therefore, we believe that a major conformational change (not just a local residue shift at the gates) should take place in the protein structure when it turns from the prostaglandin transporter mode to the ATP-conductive channel mode. Thus, the 3D-structure of the channel mode of SLCO2A1 remains to be elucidated.

### Structures of the Core Molecules of Acid-Sensitive Outwardly Rectifying/Proton-Activated Anion Channel

Identification of the ASOR/PAC channel as TMEM206 (Ullrich et al., 2019; Yang et al., 2019a) has initiated a breakthrough in revealing its spatial architecture. To date, two 3D-structures are available: human TMEM206 (Ruan et al., 2020) and its ortholog of pufferfish (Deng et al., 2021). Both channels exhibited a homo-trimeric structure with a ball-shaped extracellular domain (ECD), mostly made of β-strains, connected to a slim transmembrane (TM) domain composed of two α-helixes (TM1 and TM2) from each of the three subunits (Figure 1C). Although the ECD possesses a water-accessible cavity reminiscent of an extracellular vestibule, there is a tight seal that separates it from the channel pore inside the transmembrane region. The ion-conducting path is supposed to begin in “fenestrations” (Ruan et al., 2020) at the beginnings of transmembrane region (Figure 1C), and the contribution of the “lateral openings” seen in the ECD of the pufferfish channel to the conducting pathway has also been suggested (Deng et al., 2021). The pore is lined by residues of TM2 of each subunit with K319 in human channel (corresponds to K320 in the pufferfish protein) as a key determinant of selectivity and outward rectification. This is justified by the charge-reversing mutation K319E that confers cation selectivity and inward rectification (Ruan et al., 2020).

Channel protonation induces a large conformational change that involves movement of the protonated H98 at the interface between TMs and ECD to an “acidic pocket” formed by three negatively charged amino acids (E107, D109, and E250) inside the ECD (Ruan et al., 2020). This movement is associated with enlargement of the channel interior. However, even the structure obtained at pH 4 (Figure 1C: black line in the graph given at the right of the lower panel) is too narrow to be conductive to anions, implying that all the reported structures were caught in closed (or inactivated) states. Thus, further studies are warranted to clarify the 3D-structure of TMEM206 in the open (or activated) state.

## Physiological Roles of Volume-Sensitive Outwardly Rectifying Anion Channel/Volume-Regulated Anion Channel, Maxi-Anion Channel, and Acid-Sensitive Outwardly Rectifying/Proton-Activated Anion Channel

### Roles in Organic Signal Release of Volume-Sensitive Outwardly Rectifying Anion Channel/Volume-Regulated Anion Channel, Maxi-Anion Channel, and Acid-Sensitive Outwardly Rectifying/Proton-Activated Anion Channel

Volume-sensitive outwardly rectifying anion channel/Volume-regulated anion channel and Maxi-Cl may play roles in cellular release of organic signal molecules, because not only most inorganic but also some organic anions are permeable to VSOR/VRAC and Maxi-Cl, when the size of the given anionic substance is smaller than that of the pore size. VSOR-mediated conductance of glutamate (Glu) [its effective radius of 0.345 nm (Sabirov and Okada, 2005)] with P_glu_/P_Cl_ of around 0.2 was originally shown by Banderali and Roy (1992). Maxi-Cl was also shown to exhibit significant permeability not only to glutamate with P_glu_/P_Cl_ of around 0.2 but also to ATP [its effective radius of 0.57–0.58 nm (Sabirov and Okada, 2005)] with P_ATP_/P_Cl_ of around 0.09 evaluated originally by Sabirov et al. (2001). Since glutamate and ATP exert as extracellular paracrine/autocrine signals, these anion channels may provide the pathways for cellular release of glutamatergic/excitotoxic and purinergic signals, respectively. Actually, a large number of studies demonstrated VSOR/VRAC-mediated glutamate release and Maxi-Cl-mediated release of glutamate and ATP (see Reviews: Okada et al., 2018, 2021a). VSOR/VRAC-mediated ATP release was observed in some but not all types of cells (Hisadome et al., 2002; Dutta et al., 2004; Dunn et al., 2020; Furuya et al., 2021). Such cell type-dependent ATP release capability of VSOR/VRAC may be explained by the fact that ATP conductivity via VSOR/VRAC depends on the subunit composition of the LRRC8 hexamer (Gaitán-Peñas et al., 2016; Syeda et al., 2016). VSOR/VRAC was also shown to be conductive for an antioxidant glutathione (GSH; Sabirov et al., 2013; Friard et al., 2019) with P_GSH_/P_Cl_ of around 0.1 and for an anticancer drug cisplatin (Planells-Cases et al., 2015). Whether or not ASOR/PAC also significantly participates in organic solute release and/or uptake awaits future study for verification.

### Roles in Regulatory Volume Decrease of Volume-Sensitive Outwardly Rectifying Anion Channel/Volume-Regulated Anion Channel, Maxi-Anion Channel, and Acid-Sensitive Outwardly Rectifying/Proton-Activated Anion Channel

**FIGURE 2:**
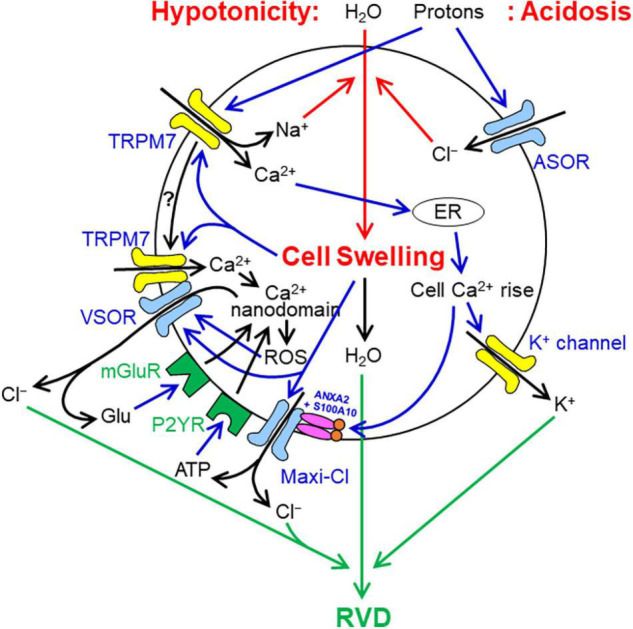
Involvements of VSOR/VRAC, Maxi-Cl, and ASOR/PAC anion channels in cell volume regulation/dysregulation under hypotonic or acidosis conditions (see in text in detail). It is noted that cell swelling results from water influx driven by osmotic gradient under hypotonic situations or by NaCl influx caused by proton-induced parallel activation of ASOR/PAC anion channels and TRPM7 cation channels under acidosis, whereas RVD is attained by water efflux driven by parallel activation of VSOR/VRAC anion channels and K^+^ channels. How TRPM7 is mobilized to physically interact with LRRC8A after osmotic cell swelling is not known. Glutamate (Glu) and ATP released from VSOR/VRAC and/or Maxi-Cl may also stimulate, in a paracrine fashion, metabotropic and ionotropic glutamate receptors and purinergic receptors expressed in neighboring cells (not drawn in this figure).

Cell volume regulation after osmotic swelling observed in most mammalian cells is called the regulatory volume decrease (RVD) which is known to be accomplished by water efflux driven by KCl efflux. The volume-regulatory KCl release was first directly shown to be predominantly attained by parallel activation of K^+^ and Cl^–^ channels in 1988 (Hazama and Okada, 1988). As shown by Numata et al. (2007b), cell swelling is initially sensed by TRPM7, which is known as a mechano-sensing cation channel in many cell types (Numata et al., 2007a; Liu et al., 2015; Won et al., 2018; Zhao R. et al., 2019; Starostina et al., 2021), and thereby causes Ca^2+^ influx. This swelling-triggered Ca^2+^ entry may trigger ryanodine-sensitive Ca^2+^-induced Ca^2+^ release from ER, thereby leading to sustained intracellular free Ca^2+^ rise (Hazama and Okada, 1990), and then Ca^2+^-activated volume-regulatory K^+^ channels (Figure 2).

Volume-regulatory pathways for Cl^–^ are mainly provided by VSOR/VRAC (Nilius et al., 1997; Okada, 1997). Molecular evidence for an involvement of LRRC8A in RVD was provided by the inhibitory effect of its gene silencing (Qiu et al., 2014; Formaggio et al., 2019) and deletion (Voss et al., 2014; Friard et al., 2017; Kang et al., 2018; Trothe et al., 2018; Green et al., 2020). Swelling-activated VSOR/VRAC activity was recently shown to be doubly supported by TRPM7 first through molecular expression of LRRC8A depending on the maintenance of steady-state Ca^2+^ influx via this cation channel and second through stabilizing plasmalemmal LRRC8A expression depending on the physical protein-protein interaction with the *C*-terminal domain of TRPM7 (Numata et al., 2021). Activation of VSOR/VRAC is known to be triggered, even in the absence of cell swelling, by ROS production controlled by Ca^2+^ nanodomains caused by stimulation of GPCRs such as purinergic P2Y receptor (P2YR; Akita et al., 2011), bradykinin receptor (Akita and Okada, 2011) and metabotropic glutamate receptor (mGluR; Akita and Okada, 2014). Thus, ATP released via Maxi-Cl and glutamate released via Maxi-Cl and VSOR/VRAC may enhance, in an autocrine fashion, VSOR/VRAC activity initially triggered by TRPM7-induced Ca^2+^ rise upon cell swelling (Figure 2).

Maxi-anion channel may also, at least in part, provide the volume-regulatory pathway for Cl^–^ (Falke and Misler, 1989). However, molecular evidence for an involvement of Maxi-Cl/SLCO2A1 in RVD remains to be missing. The mechanisms for activation of Maxi-Cl induced by cell swelling involve cytosolic free Ca^2+^ rise through Ca^2+^ binding to S100A10 (Islam et al., 2020) and also may involve mechanosensitivity of Maxi-Cl (Schwiebert et al., 1994), as schematically illustrated in Figure 2. Maxi-Cl activation is also shown to be induced by tyrosine de-phosphorylation (Toychiev et al., 2009) at Tyr23 of ANXA2 (Islam et al., 2020).

### Roles in Cell Volume Dysregulation Coupled to Cell Death Induction of Volume-Sensitive Outwardly Rectifying Anion Channel/Volume-Regulated Anion Channel, Maxi-Anion Channel, and Acid-Sensitive Outwardly Rectifying/Proton-Activated Anion Channel

In contrast to VSOR/VRAC and Maxi-Cl, ASOR/PAC rather plays a cell swelling-inducing but not volume-regulatory role. Acidosis induces lengthy cell swelling (Wang et al., 2007; Sato-Numata et al., 2014), because extracellular protons directly activate not only ASOR/PAC but also TRPM7 under acidotic conditions (Numata et al., 2019). Prolonged acid-induced activation of TRPM7 causes Na^+^ influx and cell depolarization which drives Cl^–^ influx via ASOR/PAC, thereby causing cell swelling (Figure 2).

Persistent activation of ASOR/PAC and VSOR/VRAC plays pathophysiological roles in induction of the necrotic volume increase (NVI) under acidotoxic, lactacidotoxic, excitotoxic, and hypoxic/ischemic conditions as described in recent review articles (Okada et al., 2021a,b). A positive inotropic role of ATP released via Maxi-Cl in response to ischemia-reperfusion in the heart was reported in a recent study (Matsuura et al., 2021). VSOR/VRAC activity also plays pathophysiological roles in induction of apoptotic cell death under stimulation with apoptosis inducers in a threefold manner, as described in other review articles (Okada et al., 2019a,2021a); first ROS-mediated activation of VSOR/VRAC induces persistent cell shrinkage called the apoptotic volume decrease (AVD; Maeno et al., 2000; Shimizu et al., 2004; Planells-Cases et al., 2015), second VSOR/VRAC activation mediates GSH release (Sabirov et al., 2013), thereby contributing to intracellular GSH depletion, and third VSOR/VRAC serves as the entry pathway for some anticancer drugs such as cisplatin (Planells-Cases et al., 2015) which can kill cancer cells by apoptosis (Ise et al., 2005; Sørensen et al., 2014). It is also noted that downregulation of VSOR/VRAC activity (Lee et al., 2007) and LRRC8A expression (Planells-Cases et al., 2015; Sørensen et al., 2016a,b; Thorsteinsdottir et al., 2016) is associated with acquisition of cisplatin resistance by cancer cells.

## Perspective

As outlined in Table 1 with question marks, there remain many questions concerning molecular, biophysical and physiological properties of these three types of anion channels for future studies. Also, it is still unknown about actual molecular mechanisms including intracellular signaling pathways for activation of these anion channels. Although cell swelling-induced activation of VSOR/VRAC is now known to be mediated by TRPM7, it is not elucidated how VSOR/VRAC channels are activated by reduced ionic strength. Likewise, how cell swelling activates Maxi-Cl/SLCO2A1 channel activity remain to be elucidated, whereas its activation induced by tyrosine dephosphorylation and intracellular Ca^2+^ rise was shown to be mediated by the ANXA2-S100A10 complex (Islam et al., 2020). The molecular mechanisms and intracellular signaling for ASOR/PAC channel activation are totally unknown. Furthermore, it should be reacknowledged that the exact 3D-structures have not as yet been determined for actual VSOR/VRAC channels formed by heteromers of LRRC8A *plus* LRRC8C/D/E, for the Maxi-Cl channel (but not prostaglandin transporter) conformation of SLCO2A1, and for the open/activated (but not closed/inactivated) state of ASOR/PAC/TMEM206 channels.

Physiological and pathophysiological roles played by the three discussed chloride channels in various cellular processes suggest that their dysfunctions would result in human diseases. Indeed, a number of human disease-associated mutations in genes encoding LRRC8A (see Review: Vaeth and Feske, 2018) and SLCO2A1 (see Reviews: Madruga Dias et al., 2014; Stumpff, 2018; Nakanishi et al., 2021) as well as by association of cancer progression with TMEM206 expression (Zhao J. et al., 2019; Zhang et al., 2020) have been reported. Global knockout of *Lrrc8a* in mice exhibits increased prenatal/postnatal mortality as well as a wide variety of disordered development of tissues and organs (Kumar et al., 2014). Also, a number of disorders are found in recent studies using specific cell- or tissue-targeted *Lrrc8a* knockout mice. For example, impaired glucose tolerance and insulin resistance are caused by conditional knockout of *Lrrc8a* expressing in adipocytes (Zhang et al., 2017), pancreatic β cells (Kang et al., 2018), and skeletal muscle cells (Kumar et al., 2020); male infertility by germ cell-specific *Lrrc8a* disruption (Lück et al., 2018); angiotensin-II-stimulated hypertension by endothelial-targeted *Lrrc8a* knockout (Alghanem et al., 2021); and brain hyperexcitability coupled to astrogliosis by brain-specific *Lrrc8a* disruption (Wilson et al., 2021). However, it is not known whether these dysfunctions are caused by disordered anion-conductive activities of LRRC8A or non-conductive protein-protein interactions. On the other hand, ischemia-induced brain infarct was found to be partially protected not only by astrocyte-specific *Lrrc8a* knockout presumably by suppressing excitotoxicity (Yang et al., 2019b) but also by neuron-targeted *Lrrc8a* disruption presumably through inhibition of apoptosis induction (Zhou et al., 2020). Studies with *Slco2a1* knockout mice suggested that SLCO2A1 is involved in promotion of colon cancer (Nakanishi et al., 2017), bleomycin-induced fibrosis (Nakanishi et al., 2015), and LPS-induced febrile responses (Nakamura et al., 2018). Although these effects have been interpreted by prostaglandin transporting activity of SLCO2A1, it is now required to reexamine the possible therein involvements of Maxi-Cl channel activity of SLCO2A1. It should be noted that episodic ataxia caused by dysfunction of glutamate transporter-associated chloride channels (Kovermann et al., 2020) further emphasizes pathological roles of dual transporter/channel proteins. *Tmem206* knockout mice were also found to exhibit partially protective effects against ischemic brain injury caused by permanent middle cerebral artery occlusion (pMCAO) presumably by reducing acidotoxicity (Yang et al., 2019b).

An obvious and straightforward way to correct the impaired channel functions (either inherited or acquired) is pharmacological up- or down-regulation of functional activity of the VSOR/VRAC, Maxi-Cl, and ASOR/PAC channels and/or their expression. The most selective inhibitor of VSOR/VRAC, DCPIB, has been shown to exert neuroprotective (Zhang et al., 2008; Alibrahim et al., 2013; Han et al., 2014; Wong et al., 2018) and cardioprotective (Xia et al., 2016; Wang et al., 2017) actions in animal models. Other known conventional Cl^–^ channel blockers were also shown to suppress ischemia/reperfusion-induced apoptotic cell death in cardiomyocytes (Wang et al., 2005) and excitotoxic necrotic cell death in cortical neurons and brain slices (Inoue and Okada, 2007) by inhibiting VSOR/VRAC activity. Inhibitory effects of the plant flavonoids (Xue et al., 2018; Rustamova et al., 2019), tannins (Tsiferova et al., 2019) and polyphenol gossypol (Chorieva et al., 2021) may provide a clue to discover new, effective and safe modulators targeting VSOR/VRAC. Some conventional Cl^–^ channel blockers were also found to diminish acidotoxic necrotic cell death in HeLa cells (Wang et al., 2007) and cortical neurons (Sato-Numata et al., 2014) by inhibiting ASOR/PAC activity. The pharmacological targeting of Maxi-Cl and ASOR/PAC awaits intensive studies and search among natural and synthetic compounds. More sophisticated and precise ways to manipulate the channels at the level of whole organism, such as RNAi- or CRISPR/Cas9-mediated downregulation of the expression of the channel core/pore proteins, their regulatory subunits/proteins, or the transcription factors for these genes could be considered as perspective tools for treatments of VSOR/VRAC, Maxi-Cl, and ASOR/PAC channelopathies and of their dysregulated activities under pathological conditions.

In conclusion, the core molecules of three types of volume-related anion channels, VSOR/VRAC, Maxi-Cl, and ASOR/PAC, were identified in the 2010’s at last. After that, their 3D-sturctures, regulatory subunit/partner molecules, functional properties and physiological roles became clarified on the basis of their molecular insights. By comparing these pieces of information to each other, it becomes apparent that there remain numbers of missing information to be unraveled. Accumulating evidence has revealed that these anion channels are associated not only with essential physiological cell functions but also with pathological situations involving their capability of cell volume regulation/dysregulation and organic signal release.

## Author Contributions

YO and RS wrote the manuscript with input from PM, TN, and KS-N. RS, PM, and KS-N prepared the figures. KS-N and PM prepared the references. All authors contributed to the article and approved the submitted version.

## Conflict of Interest

The authors declare that the research was conducted in the absence of any commercial or financial relationships that could be construed as a potential conflict of interest.

## Publisher’s Note

All claims expressed in this article are solely those of the authors and do not necessarily represent those of their affiliated organizations, or those of the publisher, the editors and the reviewers. Any product that may be evaluated in this article, or claim that may be made by its manufacturer, is not guaranteed or endorsed by the publisher.
